# On the Use of Iodide of Potassium in Chronic Lead Poisoning

**Published:** 1849-12

**Authors:** 


*On the Use of Iodide of Potassium in Chronic Lead Poisoning.* By M. Melsens.—The treatment proposed by MM. Melsens and Natalis Guillott rests on this principle: to render soluble the metallic compounds which the system would retain, by associating them with a body which the system most readily eliminates. This point of view has already been realized: first, by means of the property possessed by all the insoluble compounds formed by salts of mercury and the substances which are met with in the system, to become soluble in iodide of potassium; secondly, relying upon the facility and rapidity with which this system eliminates the iodide of potassium, it has been admitted by analogy that the compounds of lead retained by the system would most probably be dissolved and eliminated by iodide of potassium.

In the present communication, M. Melsens mentions several well authenticated cases of recovery from poisoning by preparations of lead. All the patients treated with iodide of potassium were cured. M. Melsens shows clearly that neither sulphuric acid, nor the sulphates can be considered as remedial agents in chronic diseases arising from handling preparations of lead, whilst the sulphate of lead is a poison sufficiently virulent to destroy animals in a few weeks. Dogs never resist its action longer than one month, and some die in a few days. When sulphate of lead and iodide of potassium are conjointly administered to a dog, no morbid effect is produced within the time necessary to kill a dog to which the sulphate alone was administered.

M. Melsens says that, if a very strong dose of iodide of potassium be administered in the first case to a dog suffering from disease arising from the administration of the sulphate, carbonate, or iodide of lead, he quickly dies—but that if on the contrary, we commence by giving small quantities of iodide of potassium at a time, and gradually increase the dose, the animal is cured in a very short time. The doses of iodide of potassium which kill a dog laboring under the effects of lead, produce no action on a healthy dog. M. Melsens also relates several cases of complete cure who he obtained by following the same plan of treatment with persons affected with tremor from working on mercurial preparations. One of these was completely cured without ever discontinuing his regular work. The mercury came away in the urine, and was found in the state of the iodide: it was impossible to find any trace of the mercury in the urine of the patient after his recovery.

The result of the facts established in this communication is that, by means of the treatment with iodide of potassium, the cure of chronic poisoning by lead or mercury is not obtained until after acute poisoning has first taken place, which acute stage the medical man is fully able to direct, according to the strength of his patient, but which ought to be an object of the most scrupulous attention on his part. The experiments also prove that, although certain medicinal preparations have an action of their own, yet they also act through the medium of substances which they find in the animal economy.

[We do not find that iodide of potassium exerts any well-marked solvent action on iodide of lead, although it very readily dissolves the iodide of mercury. It would have been most satisfactory if, before assigning the recovery of these cases to this solvent action of iodide of potassium, the experimentalist had distinctly proved that lead was thus carried out by these secretions more rapidly than when the iodide was not exhibited. Until this had been shown, the conclusion respecting the curative properties of this salt is not warranted by the facts. It has been recently tried in some well marked cases, and has signally failed.—Ed. Med. Gaz.]—*Med. Gaz.*

